# Explaining computation of predictive values: 2 × 2 table versus frequency tree. A randomized controlled trial [ISRCTN74278823]

**DOI:** 10.1186/1472-6920-4-13

**Published:** 2004-08-10

**Authors:** Anke Steckelberg, Andrea Balgenorth, Jürgen Berger, Ingrid Mühlhauser

**Affiliations:** 1 Unit of Health Sciences and Education, University of Hamburg, Martin-Luther-King Platz 6, 20146 Hamburg, Germany; 2 Institute of Mathematics and Computer Science in Medicine, University Hospital Hamburg, Martinistr. 52, 20246 Hamburg, Germany

## Abstract

**Background:**

Involving patients in decision making on diagnostic procedures requires a basic level of statistical thinking. However, innumeracy is prevalent even among physicians. In medical teaching the 2 × 2 table is widely used as a visual help for computations whereas in psychology the frequency tree is favoured. We assumed that the 2 × 2 table is more suitable to support computations of predictive values.

**Methods:**

184 students without prior statistical training were randomised either to a step-by-step self-learning tutorial using the 2 × 2 table (n = 94) or the frequency tree (n = 90). During the training session students were instructed by two sample tasks and a total of five positive predictive values had to be computed. During a follow-up session 4 weeks later participants had to compute 5 different tasks of comparable degree of difficulty without having the tutorial instructions at their disposal. The primary outcome was the correct solution of the tasks.

**Results:**

There were no statistically significant differences between the two groups. About 58% achieved correct solutions in 4–5 tasks following the training session and 26% in the follow-up examination.

**Conclusions:**

These findings do not support the hypothesis that the 2 × 2 table is more valuable to facilitate the calculation of positive predictive values than the frequency tree.

## Background

Diagnostic procedures are increasingly expected by consumers to ensure their health; "certainty" has become a product [1]. Assuming that test results are certain, only a minority is aware about false positive and false negative alarms. Previous research has shown that even physicians have great difficulties in estimating the positive predictive values of diagnostic tests [2-4]. One study reported that 95 out of 100 physicians estimated the positive predictive value of screening mammography to be between 70–80% rather than 7.8% [2]. Similar results were reported for AIDS counselors for low-risk clients. The majority of counselors assured that false positives would never occur and half of the counselors incorrectly assured that if a low-risk person tests positive, it is absolutely certain (100%) that he or she is infected with the virus [5]. An incorrect probability judgment may result in unnecessary tests or pseudo certainty. Therefore, the understanding, presentation and communication of test quality are a challenge for both: lay people and professionals.

Involving lay people in decision making on diagnostic procedures requires a basic level of statistical thinking. Help for computing Bayesian inference is needed. Statistical thinking can be enhanced by representing statistical information in terms of natural frequencies rather than probabilities [6,7]. This is explained by the evolution of the human reasoning system. Gigerenzer proposed that human reasoning is algorithms designed for information that comes in a format that was present in the "environment of evolutionary adaptiveness" [8]. Human reasoning processes are adapted to natural frequencies. Also Bayesian computations are easier when the information is communicated this way.

**Figure 1:**
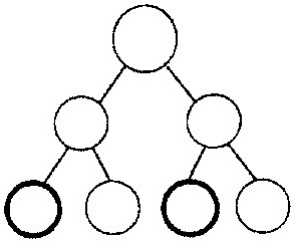
Frequency tree.

In cognitive psychology the frequency tree is used as visual help for the representation of frequencies, a variant of a tree structure often used in decision analysis to teach computing the positive predictive value the simple way (Figure 1) [4]. This format allows a multistage presentation of the numerical information and demonstrates the reasoning process.

**Figure 2:**
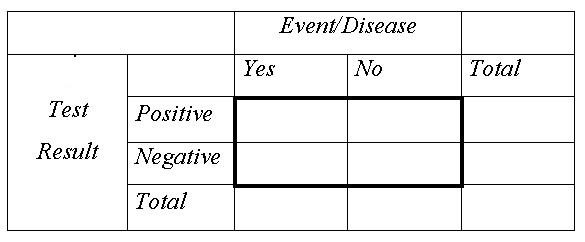
2 × 2 table.

In contrast, in medical science the 2 × 2 table is the standard method to teach computing predictive values (Figure 2) [9,10]. In addition, the 2 × 2 table is used for other calculations, e.g. odds ratios or relative risks [9].

In the present study, we compare the two visual helps in non-medical students. We hypothesized that the 2 × 2 table is more eligible than the frequency tree to facilitate correct answers in tasks of calculations of positive predictive values 4 weeks after an initial training-session. We also describe students' ability to calculate positive predictive values, analyzing the transfer of the numerical information into the visual help and the correct computation.

## Methods

### Participants

We approached 238 students without prior statistical training to recruit the necessary 184 students who agreed to participate. (See power calculation below) Students attending the University of Hamburg (health sciences, biology and sports), a vocational college (health and nursing) or taking part in an in-service training (nursing and public health) were informed about the timing and content procedure of the study during their courses.

### Procedure

The study was carried out between October 2000 and July 2001 and consisted of two supervised sessions lasting about 1 h each. The recruited 184 students were randomly assigned either to the frequency tree group (n = 94) or to the 2 × 2 table group (n = 90) using blocked randomization in blocks of 10. Concealed allocation based on computer-generated random numbers was done by an external person. In addition, the external person prepared sealed envelopes for both sessions including the tutorial with the tasks and a questionnaire for survey of age, gender, years of school, mark in mathematics and social state. The training consisted of a written step-by-step self-learning tutorial (Additional file 1, 2, 3). The participants had to compute 5 positive predictive values in each session. The tutorial and tasks followed the recommendations for the presentation of numerical information [4]. Participants were asked to reveal how they achieved their solutions. Participants were allowed to use a pocket calculator. Correct results were presented and discussed after each session.

In the follow-up examination participants were again asked to solve 5 different diagnostic problems of similar level of difficulty but without having the tutorial instructions at their disposal (Additional file 4,5,6). Participants who missed the date were repeatedly contacted by letter, phone or e-mail. Efforts were discontinued after 4 weeks.

### Assessing performance

#### Correct solution of the tasks

A solution was classified correct, when the documented positive predictive value was equivalent to the correct solution rounding up or down to the next full percentage point. If a participant used the correct computation (correct positives divided by all positives) but made a calculation error either in the transfer of the numerical information into the visual help or within the division, we ignored calculation errors. Whenever a different computation such as rule of three – a mechanical method for solving proportions – was used or the calculation protocol was missing the rounded solutions were classified likewise as correct by congruence. If the protocol indeed showed that a correct rounded solution resulted from an incorrect computation such as positive predictive value = correct positives / false positives the answer was classified as incorrect. Tasks that had not been worked on were also classified incorrect.

#### Correct transfer

To evaluate the usefulness of the different visual helps, we evaluated the ability of correct transfer of the numerical information into the charts. A transfer was classified as correct, when the numerical information of the problems was inserted into the gaps provided. It was sufficient to insert the relevant values for the computation, calculation errors were ignored.

#### Correct computation

The computation was classified as correct Bayesian approach when the following computation was used: positive predictive value = correct positives / (correct positives + false positives) or positive predictive value = correct positives / all positives. The computation was classified as Non-Bayesian approach when the computation was used with false values. Other computations were classified as other strategies.

### Statistical power and analyses

Table 1 shows the hypothesized distribution of correct answers within the different categories as primary outcome measure between the two study groups (Table 1). By using the Wilcoxon (Mann-Whitney) rank-sum Test in a sample of 92 persons in each group (84 + 10% drop-out) the hypothesized differences are detected with a power of 80% at a 2- tailed α of 0.05. For our one-sided hypothesis that the 2 × 2 table is superior to the frequency tree the power is 88% at sample size of n1 = n2 = 80.

**Table 1:** Hypothesized distribution of correct answers after 4 weeks between the two study groups

Categories* (numbers of correct answers)	Frequency tree	2 × 2 table
0	0.40	0.30
1	0.15	0.05
2	0.15	0.05
3	0.10	0.20
4	0.10	0.20
5	0.10	0.20

* category 0 = 0 answers correct category 1–5 = 1–5 answers correct

Analysis is based on the intention-to-participate principle that includes all randomised participants as randomised. Drop outs were considered as having solved none of the positive predictive values correctly.

## Results

**Figure 3:**
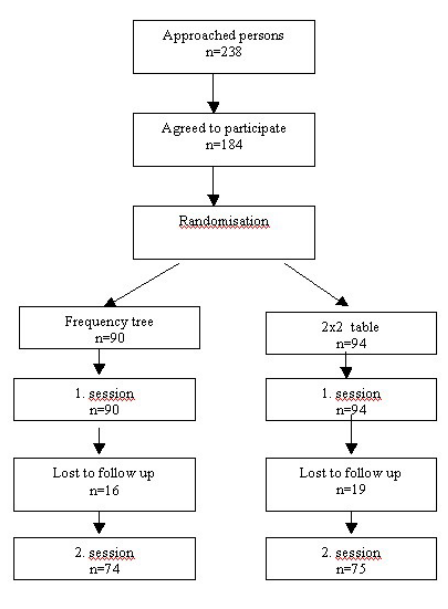
Flow of participants.

Figure 3 shows the flow of participants through the trial (Figure 3). There were 18% drop outs in the frequency tree group and 20% in the 2 × 2 table group resulting in a power of 78% for the two-sided and 86% for the one-sided hypothesis. For grouping into three categories as used for analyses the power is 81% for the two-sided and 89% for the one-sided hypothesis.

The groups were similar regarding demographic variables (Table 2).

**Table 2:** Baseline characteristics*

	Frequency tree (n = 94)	2 × 2 table (n = 90)
**Age**		
Median (range)	29 (20–54)	26 (19–51)
Missing values	3 (3)	2 (2)
**Gender**		
Male	15 (16)	20 (22)
Female	77 (82)	67 (75)
Missing values	2 (3)	3 (3)
**Years of school**		
< 10 years	1 (1)	1 (1)
10–12 years	22 (23)	19 (21)
> 12 years	68 (72)	67 (75)
Missing values	3 (3)	3 (3)
**Mark in mathematics**		
1 (highest level)	6 (6)	6 (7)
2	20 (21)	18 (20)
3	35 (37)	32 (36)
4	14 (15)	17 (19)
5 (lowest level)	5 (5)	9 (10)
Missing values	14 (15)	8 (9)
**Group**		
University of Hamburg	59 (63)	55 (61)
Vocational College	14 (15)	15 (17)
Non-academic students	21 (22)	20 (22)

*Values are numbers (percentages) of participants unless stated otherwise

### Correct solutions of the tasks

Table 3 shows the solutions of both sessions with regard to the primary outcome. Within the training session 20% of participants in both groups calculated only 0–1 answers correctly; 58% (95% CI, 47%–68%) (2 × 2 table) and 59% (95% CI, 48%–69%) (frequency tree), respectively, solved 4 or 5 tasks correctly. In the follow-up examination most participants could not solve more than 0–1 tasks correctly (72% frequency tree and 67% 2 × 2 table).

**Table 3:** Numbers of correct solutions of positive predictive values*

**Category**	**Training session**		**Follow-up examination**	
	Frequency tree (n = 94)	2 × 2 table (n = 90)	Frequency tree (n = 74)	2 × 2 table (n = 75)

**0–1 ** (0–1 answer correct)	19(20)	18(20)	53 (72)	50 (67)
**2–3 ** (2–3 answers correct)	20 (21)	20 (22)	2 (3)	5 (7)
**4–5 ** (4–5 answers correct)	55 (59)	52(58)	19 (26)	20(27)

* Values are numbers (percentages) of participants

Within the category 4–5 correct answers 27% of participants (95% CI, 17%–38%) (2 × 2 table) and 26% (95% CI, 16%–37%) (frequency tree) had correct solutions. The differences between the two study groups were not statistically significant neither in the training session (p = 0.95 {0.49 one-sided}) nor in the follow-up examination (p = 0.48 {0.24} for the analysis on intention-to-participate and p = 0.61 {0.31} for the analysis on-participation (Table 3).

In addition, we analyzed every single task in terms of correct solution. In the training session 66% of all questions [(n = 309/470 (frequency tree); n = 297/450 (2 × 2 table)] were solved correctly in both groups. The amount of correct solutions decreased to 26% (n = 98/370) and 31% (n = 115/375), respectively, in the follow-up examination. Differences between groups were not statistically significant (Table 4).

**Table 4:** Analysis of each task regarding correct solutions, transfer of numerical information and Bayesian computations*

	**Correct solution**			
	Training session		Follow-up examination	

	frequency tree (n = 94)	2 × 2 table (n = 90)	frequency tree (n = 74)	2 × 2 table (n = 75)
Task A	67 (71)	66 (73)	18 (24)	25 (33)
Task B	63 (67)	64 (71)	22 (30)	24 (32)
Task C	69 (73)	63 (70)	19 (26)	23 (31)
Task D	67 (71)	54 (60)	21 (28)	23 (31)
Task E	43 (46)	50 (56)	18 (24)	20 (27)
	**Correct transfer**			

	Training session		Follow-up examination	

	frequency tree (n = 94)	2 × 2 table (n = 90)	frequency tree (n = 74)	2 × 2 table (n = 75)
Task A	84 (89)	79 (88)	53 (72)	57 (76)
Task B	83 (88)	78 (87)	52 (70)	57 (76)
Task C	71 (76)	65 (72)	45 (61)	53 (71)
Task D	73 (78)	67 (74)	42 (57)	49 (65)
Task E	54 (57)	53 (59)	42 (57)	48 (64)
	**Correct Bayesian Computation**			

	Training session		Follow-up examination	

	frequency tree (n = 94)	2 × 2 table (n = 90)	frequency tree (n = 74)	2 × 2 table (n = 75)
Task A	62 (66)	59 (66)	13 (18)	18 (24)
Task B	60 (64)	58 (64)	17 (23)	18 (24)
Task C	70 (75)	60 (67)	15 (20)	15 (20)
Task D	69 (73)	52 (58)	16 (22)	17 (23)
Task E	46 (49)	44 (49)	15 (20)	15 (20)

* Values are numbers (percentages) of tasks

### Correct transfer

Transfer of the numerical information into the visual help in the training session could be managed in 78% (n = 365/470 frequency tree) and 76% (n = 342/450 2 × 2 table) of the tasks. In the follow-up examination in 63% (n = 234/370) and 70% (n = 264/375), respectively, the information was correctly transferred into the visual helps (Table 4).

### Correct computation

The application of the Bayesian computation in the training session was correctly used in 65% (n = 307/470 frequency tree) and in 61% (n = 273/450 2 × 2 table). In the follow-up examination 21% (n = 76/370) and 22% (n = 83/375), respectively, used correct Bayesian computation (Table 4).

### Incorrect Bayesian approaches

Table 5 shows the commonly used incorrect Bayesian approaches which lead to incorrect solutions of the tasks (Table 5).

**Table 5:** The commonly used incorrect Bayesian approaches*

	Training session			Follow-up examination		
	total	Frequency tree	2 × 2 table	total	Frequency tree	2 × 2 table

correct positive rate/ false positive rate	41	26 (63)	15 (37)	16	11 (69)	5 (31)
disease yes / all positives	14	7 (50)	7 (50)	37	20 (54)	17 (46)
correct positives / disease yes	11	6 (55)	5 (45)	22	11 (50)	11 (50)
all positives / total	4	4 (100)	0 (0)	14	6 (43)	8 (57)
all positives / 100	0	0 (0)	0 (0)	6	6 (100)	0 (0)
disease yes / correct positives	4	1 (25)	3 (75)	1	1 (100)	0 (0)
all positives/ correct positives	4	0 (0)	4 (100)	5	5 (100)	0 (0)
not identified	23	13 (57)	10 (43)	29	14 (48)	15 (52)
total	101	57 (56)	44 (44)	130	74 (57)	56 (43)

* Values are numbers (percentages) of incorrect Bayesian approaches.

## Discussion

Differences between the 2 × 2 table and the frequency tree groups were neither meaningful nor statistically significant with regard to the primary outcome measure of correct calculation of the positive predicted values. In the training session the majority of participants were able to calculate the positive predictive value of all tasks correctly. In the reexamination after 4 weeks the proportion of participants with solutions of all tasks decreased to 26% in both groups. The transfer of the numerical information into the visual helps was comparable between the two sessions. However, participants had major difficulties in applying the correct computation as a precondition of a correct solution.

In all our tasks we have used frequency formats following the recommendation of Gigerenzer & Hoffrage [4]. In those earlier studies the frequency tree without caption has been used and we adopted this format of the frequency tree in our study. However, in more recent studies a captioned frequency tree has been used [11]. Therefore, we cannot exclude that when comparing the 2 × 2 table with a captioned frequency tree the results might be different.

Our study is the first that has compared the two visual helps 2 × 2 table and frequency tree. Previous studies have concentrated on teaching methods using either one of the visual helps or both in combination [4,12]. These previous studies addressed different target groups, mainly medical students and physicians and focused different questions. In contrast, we addressed non-medical students without prior statistical knowledge as a first approach to lay people. Therefore, the overall results of our study are difficult to compare to previous publications.

The primary aim of our study was not to investigate different teaching methods for computing predictive values. We have tried to apply the most appropriate method according to actual research at the initiation of the study. However, overall performance of our students was poor. In the training session 58% of participants were able to calculate the positive predictive value of 4 or 5 tasks correctly. In the follow-up examination after 4 weeks the proportion of correct solutions in 4 or 5 tasks decreased to 26%. In addition, after 4 weeks participants had major difficulties in applying the correct computation as a precondition of a correct solution whereas there was only a minor deterioration with respect to the transfer of the numerical information into the visual helps.

A recent study used a computerized tutorial programme to teach Bayesian inference [11]. Within the study carried out in a rather small sample of mostly medical students, the role of the graphical aids captioned frequency tree presenting data as natural frequencies versus probability tree presenting data as probabilities in teaching Bayesian inference was explored. After 3 month participants who used the frequency tree reached 100% Bayesian solutions compared with 57% of participants using the probability tree. The authors hypothesized that it is much more important whether the proper representation is used than which graphical aid is applied [11]. Kurzenhauser & Hoffrage studied the effects of a classroom tutorial using both visual helps to teach Bayesian reasoning [12]. They achieved 47% correct answers after 2 months. Participants of the study were medical students in their second and third semester.

Generalisability of the results with respect to the overall correct solutions of our study may be limited by the prevalent innumeracy that has lately been ascertained for Germany within the OECD Programme for international student assessment (PISA). Mathematics literacy was stated to be poor in Germany especially in girls [13]. A high percentage of participants in our study were women which corresponds to the distribution of students. Transferring the self-learning tutorial to people without general qualification for university entrance would probably result in an even lower amount of correct solutions.

## Conclusions

In conclusion, our findings do not support the hypothesis that the 2 × 2 table is more valuable to facilitate the calculation of positive predictive values than the frequency tree. Regardless which visual help is used there is a need for improvement of teaching methods to approach lay people who want to participate in medical decision making.

## Competing interests

None declared.

## Authors' contributions

AS as the principal investigator planned and performed the study analysed the data and wrote the paper. AB contributed to planning and performance of the study. JB calculated the power of the study and carried out the statistical analysis of data. IM contributed to all parts of the study. All authors read and approved the final manuscript.

## Pre-publication history

The pre-publication history for this paper can be accessed here:



## Supplementary Material

Additional File 1 Original questionnaire used in the 2 × 2 table group in the 1. session in German language. Click here for file

Additional File 2 Original questionnaire used in the frequency tree group in the 1. session in German language. Click here for file

Additional File 3 Tasks used in the questionnaires of the training session in English language. Click here for file

Additional File 4 Original questionnaire used in the 2 × 2 table group in the 2. session in German language. Click here for file

Additional File 5 Original questionnaire used in the frequency tree group in the 2. session in German language. Click here for file

Additional File 6 Tasks used in the questionnaires of the follow-up examination in English language. Click here for file
