# Supplementary material for: Explaining computation of predictive values: 2 × 2 table versus frequency tree. A randomized controlled trial [ISRCTN74278823]
Source: BMC Med Educ. 2004 Aug 10;4:13. doi: 10.1186/1472-6920-4-13 (PMC514564; doi:10.1186/1472-6920-4-13)
Supplement: Additional File 2 — Original questionnaire used in the frequency tree group in the 1. session in German language. [file 1472-6920-4-13-S2.pdf]

Bitte lesen Sie die folgende Musteraufgabe konzentriert durch.

Musteraufgabe I

Mit dem Ziel der Früherkennung von Brustkrebs werden Frauen angehalten, ab einem bestimmten Alter regelmäßig eine Röntgenuntersuchung der Brust durchführen zu lassen, selbst wenn keine Symptome vorliegen. Für symptomfreie Frauen im Alter zwischen 40 und 50 Jahren, die im Rahmen einer Reihenuntersuchung eine Mammographie durchführen lassen, liegen folgende Informationen vor:

Von je 1000 dieser Frauen haben 10 Brustkrebs. Von diesen 10 Frauen, die Brustkrebs haben, erhalten 8 einen positiven [1] Mammographie-Befund. Von den restlichen 990 Frauen, die keinen Brustkrebs haben, erhalten dennoch 99 einen positiven Mammographie-Befund.

Stellen Sie sich nun vor, Sie hätten sich ebenfalls dieser Untersuchung unterzogen und als Ergebnis einen verdächtigen Befund. Mit welcher Wahrscheinlichkeit liegt bei Ihnen Brustkrebs vor?

ⓘ Informationen zur Lösung folgen auf der nächsten Seite...

[1] An dieser Stelle sei darauf hingewiesen, dass in der Medizin ein positives Ergebnis gleichbedeutend ist mit einem pathologischen Befund. Ein positives Ergebnis besagt demnach, dass mit der Untersuchung etwas gefunden wurde, das vom Normalen abweicht.
Entsprechend umgekehrt verhält es sich mit einem negativen Ergebnis. Letzteres besagt, dass mit der Untersuchung nichts gefunden wurde, das vom Normalen abweicht.

Folgen Sie bitte nun den Ausführungen zur Darstellung der Zahlen mit den Natürlichen Häufigkeiten
Die Berechnung des Risikos wird anschließend vorgestellt.

Darstellung mit den Natürlichen Häufigkeiten

Abb.1

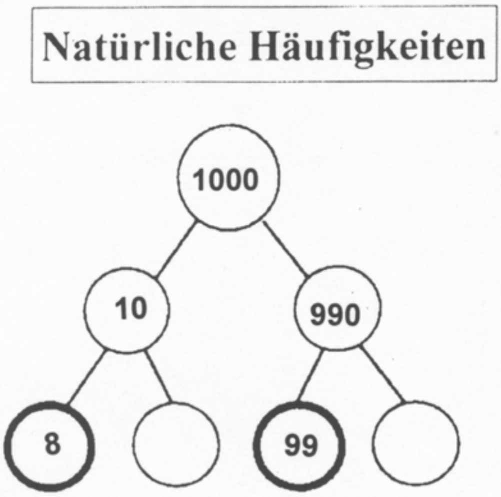

Übertragen Sie bitte nacheinander die Werte/Häufigkeiten aus dem Fallbeispiel in die dafür vorgesehenen Felder des Schaubildes.
Gehen Sie nun die Graphik noch einmal durch. **1000** Frauen haben sich der Untersuchung unterzogen. **10** von ihnen haben Brustkrebs. Bei **8** von diesen **10** „Kranken" wird die Erkrankung durch den Mammographie-Befund korrekt festgestellt. Von den restlichen **990** Frauen, die keinen Brustkrebs haben („Gesunde"), erhalten **99** fälschlicherweise einen positiven Befund.(Abb.1)

Abb.2

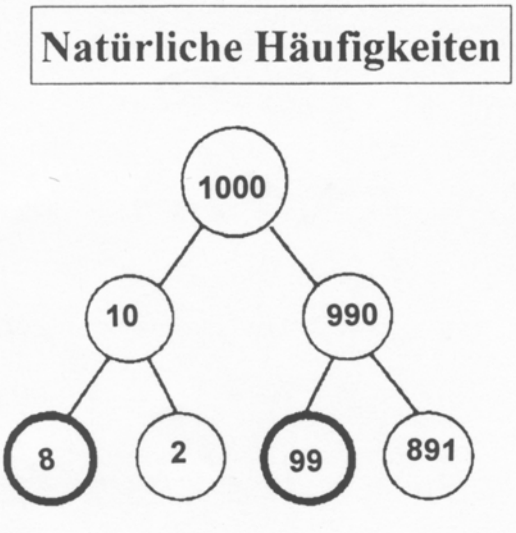

Ergänzt man nun die fehlenden Werte durch Addition /Subtraktion kann man zusätzlich ableiten:
- **2** der „Kranken" hatten fälschlicherweise einen negativen Befund
- bei **891** der „Gesunden" war der Test auch tatsächlich negativ. (Abb.2)

1

Wie groß ist die Wahrscheinlichkeit, dass bei einem positiven Mammographiebefund tatsächlich Brustkrebs vorliegt?

Berechnung des Risikos:

Insgesamt hatten nach der Untersuchung **8+99 = 107** Frauen einen positiven Befund.

Nur **8** von diesen **107** Frauen sind aber tatsächlich erkrankt = **8/107.** Die Wahrscheinlichkeit, daß Sie mit einem positiven Test zu den 8 tatsächlich Kranken Frauen gehören liegt demnach bei

8/107 = 8:107 = 0.075, das entspricht einem Prozentwert von **0,075x100 = 7,5%**

Fallbeispiel A

Etwa 0,5% der Menschen zwischen 60 und 69 Jahren leiden an Darmkrebs. Das entspricht 500 von 100.000 Menschen. Bei einem gebräuchlichen Test, der zur Feststellung von Blut im Stuhl für die Frühdiagnose des Darmkrebses eingesetzt wird (Häemoccult-Test), fällt der Test bei 250 der Erkrankten positiv aus. Von den verbleibenden 99.500 haben 9950 ebenfalls ein positives Testergebnis.

Abb.1

Natürliche Häufigkeiten

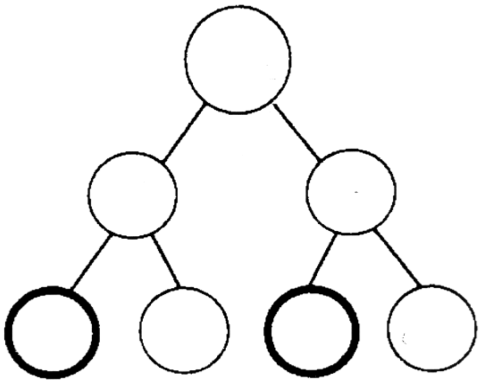

Werte/Häufigkeiten aus dem Fallbeispiel in die dafür vorgesehenen Felder des Schaubildes Abb.1. Ergänzen Sie die fehlenden Werte durch Addition/Subtraktion.

Die Wahrscheinlichkeit, bei einem positiven Testergebnis tatsächlich Darmkrebs zu haben, beträgt..........%.

Dokumentieren Sie bitte Ihre Rechenschritte.

Fallbeispiel B

Das Blut von Blutspendern wird generell u.a. auf das HIV-Virus getestet. Unter den insgesamt 20.000 Blutspendern eines großen deutschen Krankenhauses, die hinsichtlich einer möglichen HIV Infektion getestet wurden, gab es nur einen Infektionsfall. Dieser hatte auch ein positives Testergebnis. Von den 19.999 nicht infizierten Blutspendern hatten allerdings 40 ebenfalls ein positives Testergebnis.

Abb.1

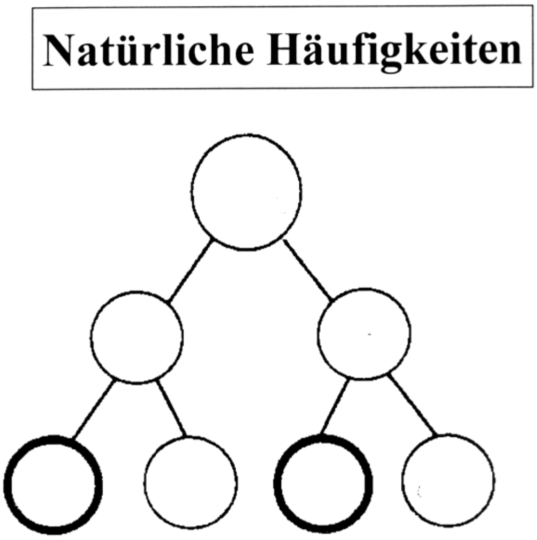

Übertragen Sie bitte nacheinander die Werte/Häufigkeiten aus dem Fallbeispiel in die dafür vorgesehenen Felder der Natürlichen Häufigkeiten (Abb.1). Ergänzen Sie der Übersicht wegen die fehlenden Werte durch Addition/Subtraktion.

Wie groß ist die Wahrscheinlichkeit, dass ein Blutspender mit einem positiven Testergebnis auch tatsächlich HIV-infiziert ist?

Die Wahrscheinlichkeit beträgt..........%.

Dokumentieren Sie bitte Ihre Rechenschritte.

Bitte lesen Sie die Musteraufgabe II konzentriert durch

Musteraufgabe II

Dieses Beispiel spiegelt Daten wieder, die ein Testverfahren charakterisieren:

Etwa 40% aller Patienten (oder 400 von 1000) mit einer Gallenkolik haben ein Gallensteinleiden. Die Abklärung der Erkrankung erfolgt durch eine Ultraschalluntersuchung, ein Verfahren das schmerzlos, risikoarm und schnell durchzuführen ist und relativ gute Ergebnisse liefert: Bei 356 von 400 Patienten mit Gallensteinen wurden die Gallensteine richtig erkannt und 582 der Patienten ohne Gallensteine, hatten ein negatives Testergebnis.

Wie hoch ist die Wahrscheinlichkeit, bei einem positiven Ultraschall-Befund auch tatsächlich Gallensteine zu haben?

ⓘ Informationen zur Lösung folgen auf der nächsten Seite...

Darstellung mit den Natürlichen Häufigkeiten

Abb. 1

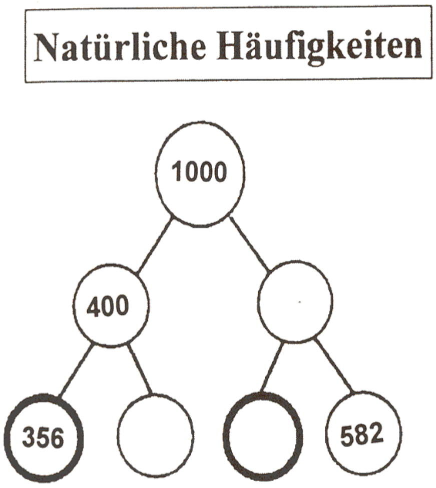

Übertragen Sie bitte nacheinander die Werte/Häufigkeiten aus dem Fallbeispiel in das Schaubild der Natürlichen Häufigkeiten Gehen Sie nun die Graphik noch einmal durch.
400 von **1000** Patienten mit Gallenkolik haben ein Gallensteinleiden.(Abb. 1). Bei **356** von ihnen, wurde dies richtig erkannt, d.h. sie hatten ein positives Untersuchungsergebnis. **582** der Patienten ohne Gallensteine hatten ebenfalls ein richtiges Untersuchungsergebnis, sie hatten korrekterweise einen negativen Befund. (Abb.1)

Abb. 2

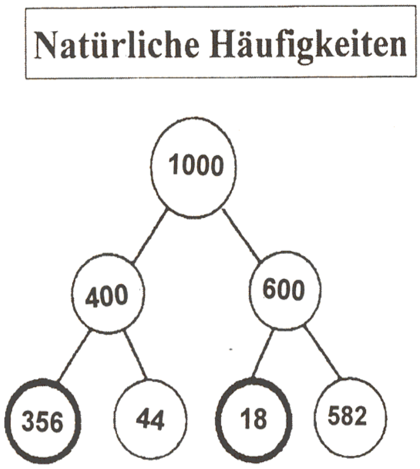

Ergänzt man nun die fehlenden Werte durch Addition /Subtraktion kann man zusätzlich ableiten:
- **44** der Patienten mit Gallensteinen hatten fälschlicherweise einen negativen Befund
- **18** der Patienten ohne Gallensteine hatten fälschlicherweise einen positiven Befund.
- Die Anzahl der Patienten ohne Gallensteine beträgt **600** (Abb. 2)

Wie hoch ist die Wahrscheinlichkeit für eine Person mit Gallenkolik bei einem positiven Ultraschall-Befund tatsächlich Gallensteine zu haben?

Berechnung des Risikos:

Insgesamt hatten nach der Untersuchung **356+18 = 374** Patienten einen positiven Befund. Nur **356** von diesen **374** Patienten haben tatsächlich Gallensteine = **356/374.**

Die Wahrscheinlichkeit, daß ein Patient mit Gallenkolik bei einem positiven Test zu den **356** mit Gallensteinen gehört, liegt demnach bei **356/374 = 356:374 = 0.9519**

Das entspricht einem Prozentwert von ca.**0.9519 x 100 = 95,19% = ca. 95%**

Fallbeispiel C

In einer Studie wurde bei 1000 Frauen ein Schwangerschaftstest durchgeführt. 20 dieser Frauen waren schwanger. Der Test war bei 20 von ihnen positiv und 975 der nicht schwangeren Frauen hatten einen negativen Test.

Abb.1

Natürliche Häufigkeiten

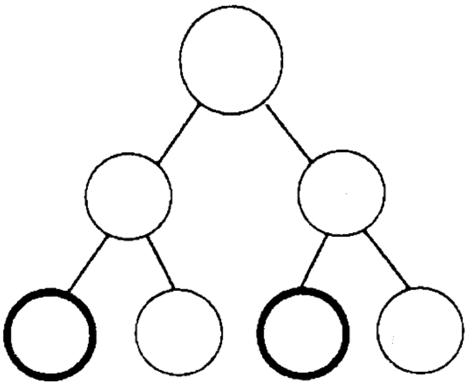

Übertragen Sie bitte nacheinander die Werte/Häufigkeiten aus dem Fallbeispiel in die dafür vorgesehenen Felder der Natürlichen Häufigkeiten (Abb.1). Ergänzen Sie die fehlenden Werte durch Addition/ Subtraktion.

Stellen Sie sich vor, eine Frau würde einen Schwangerschaftstest durchführen lassen und das Ergebnis wäre positiv. Wie groß ist die Wahrscheinlichkeit, dass sie tatsächlich schwanger ist?

Die Wahrscheinlichkeit beträgt ....%.

Dokumentieren Sie bitte Ihre Rechenschritte.

Fallbeispiel D

Es soll geklärt werden, inwieweit die Serumferritin Bestimmung als diagnostischer Test auf Eisenmangelanämie geeignet ist.

Hierzu wurden 2579 anämische Patienten untersucht. Insgesamt gab es in dieser Gruppe 809 Patienten mit Eisenmangelanämie. Bei 731 der erkrankten Patienten war das Ergebnis positiv (Serumferritin<65 mmol/l). 1500 der Patienten, die die Erkrankung nicht hatten, hatten ein negatives Testergebnis (Serumferritin>65mmol/l).

Abb.1

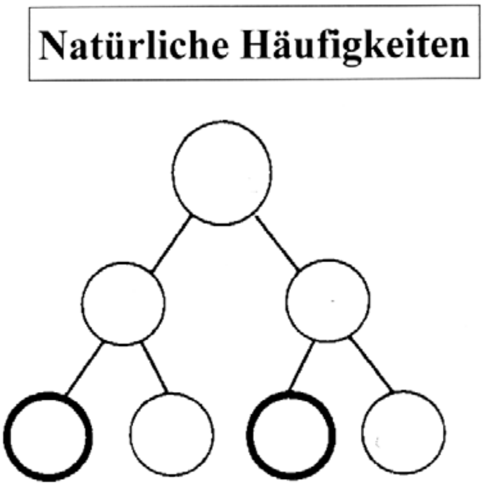

Übertragen Sie bitte nacheinander die Werte/Häufigkeiten aus dem Fallbeispiel in die dafür vorgesehenen Felder der Natürlichen Häufigkeiten (Abb.1). Ergänzen Sie die fehlenden Werte durchAddition/ Subtraktion.

Mit welcher Wahrscheinlichkeit kann ich, sofern ich an einer Anämie leide, bei einem niedrigen Serumferritin davon ausgehen, dass ein Eisenmangel der Grund für meine Anämie ist? Die Wahrscheinlichkeit beträgt ....%.

Dokumentieren Sie bitte Ihre Rechenschritte.

Bearbeiten Sie bitte das Fallbeispiel E

Fallbeispiel E

Zur Abklärung eines positiven Häemocculttests werden weitere diagnostische Verfahren nötig. Üblicherweise wird als Folgeuntersuchung eine Darmspiegelung durchgeführt wird.

Erinnern Sie sich? In unserem Beispiel A hatten 10200 Testpersonen einen positiven Haemocculttest. Bei all diesen Personen wird nun eine Darmspiegelung durchgeführt. Die Zahl der Personen mit Darmkrebs (500) bleibt. Mit der Darmspiegelung haben alle diejenigen, die die Krankheit nicht haben auch ein negatives Untersuchungsergebnis. 470 der Erkrankten haben ein positives Testergebnis.

Abb.1

Natürliche Häufigkeiten

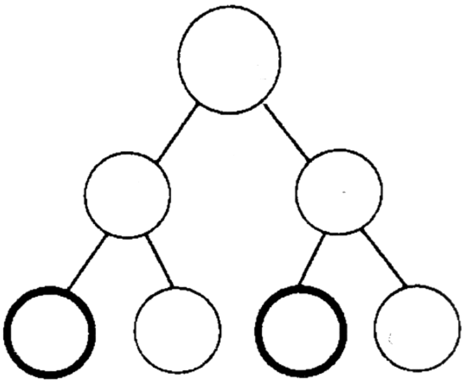

Übertragen Sie bitte nacheinander die Werte/Häufigkeiten aus dem Fallbeispiel in die dafür vorgesehenen Felder der Natürlichen Häufigkeiten (Abb.1)

Wie groß ist nun die Wahrscheinlichkeit bei einem positiven Befund bei der Darmspiegelung auch tatsächlich Darmkrebs zu haben?

Die Wahrscheinlichkeit beträgt..........%.

Dokumentieren Sie bitte Ihre Rechenschritte.
