# Supplementary material for: Explaining computation of predictive values: 2 × 2 table versus frequency tree. A randomized controlled trial [ISRCTN74278823]
Source: BMC Med Educ. 2004 Aug 10;4:13. doi: 10.1186/1472-6920-4-13 (PMC514564; doi:10.1186/1472-6920-4-13)
Supplement: Additional File 3 — Tasks used in the questionnaires of the training session in English language. [file 1472-6920-4-13-S3.pdf]

Tasks of the training session

Sample task I and task A are shown as presented in the training session of the study, for the tasks B-D only the problems are shown.

Please read the following sample task with concentration

Sample task I

Aiming at the early detection of breast cancer women are invited to have a mammography regularly, even without having symptoms. For women aged between 40 and 50 who do not have any symptoms, the following information is available:

10 of every 1,000 of these women have breast cancer. 8 of every 10 women with breast cancer will get a positive mammogram.* Out of the remaining 990 women who do not have breast cancer, 99 will still have a positive mammogram.

Imagine you have a mammography and the test result is positive. What is the probability for you to have breast cancer?

Information about the answer to the problem will follow on the next page…

*In medicine a positive test result is synonymous with a pathological diagnostic finding. A positive test result means that something differing from normal has been detected with the test.
The same applies inversely to a negative test result. The latter means that nothing differing from normal has been detected with the test.

Please follow the model of representation of numbers with the 2x2 table.

The computation of the risk will follow subsequently.

Presentation using the 2x2 table

Figure 1

		Event/Disease		
		Yes	*No*	*Total*
Test Result	*Positive*	**8**	**99**	
	Negative			
	Total	**10**	**990**	**1000**

Please transfer the information of the example successively into the provided fields of the 2x2 table.
Please go over the graphic again. **1000** women had a mammogram. **10** out of these women have breast cancer. **8** out of these **10** women get their breast cancer detected correctly. Out of the remaining **990** women who do not have breast cancer **99** incorrectly receive a positive test result. (figure 1)

Figure 2

		Event/Disease		
		Yes	*No*	*Total*
Test Result	*Positive*	**8**	**99**	**107**
	Negative	**2**	**891**	**893**
	Total	**10**	**990**	**1000**

If you complete the missing values by adding or subtraction you can derive:
2 of the women who have breast cancer have a false negative test result.
891 of the healthy women have a negative test result. (figure 2)

What is the probability of actually having breast cancer, if the test result of the mammogram is positive?

Computation of risk:

After the mammogram a total of **8+99=107** women have a positive test result.
Only **8** out of these **107** women actually have breast cancer=**8/107**. The probability that a woman with a positive test result belongs to these **8** women who actually have breast cancer is **8/107=8:107=0.075** which responds to a percentage of **0.075x100=7.5%**

Task A

About 0.5% of people aged between 60 and 69 years suffer from colorectal cancer, that means 500 out of 100,000. Using a common test to detect occult blood in the stool (FOBT), which is applied in colorectal cancer screening, 250 out of those who actually have colorectal cancer will get a positive test result. 9,950 out of the remaining 99,500 will also receive a positive test result.

Figure 1

		Event/Disease		
		Yes	*No*	*Total*
Test Result	*Positive*			
	Negative			
	Total			

Please transfer the information of the example stepwise into the provided fields.
Complete the missing values by adding or subtraction.

The probability of actually having colorectal cancer if the test result is positive is ………..%.

Please write down your calculations.

Task B

Blood tests of blood donors are generally tested on HIV. Out of a total of 20,000 blood donors of a big German hospital who are tested on a possible HIV infection there is just one HIV infection. This person has a positive test result. Out of those 19,999 blood donors who are not infected 40 also have a positive test result.

Sample task II

About 40% (or 400 out of 1,000) of all patients with biliary colic have gallstones The disease is usually diagnosed by ultrasonography, a test which is painless, fast to carry out, with low risk, providing fairly good results: 356 out of every 400 patients with gallstones, the gallstones are diagnosed correctly and 582 out of those patients without gallstones have a negative test result.

Task C

Within a study 1,000 women have a pregnancy test. 20 out of these women are actually pregnant. These 20 women have a positive test result. 975 women who are not pregnant have a negative test result.

Task D

2,579 anaemic patients are examined. A total of 809 patients have iron deficiency anaemia. 731 out of the diseased patients have a positive test result (serum ferritin < 65 mmol/l). 1,500 patients without iron deficiency anaemia have a negative test result (serum ferritin > 65 mmol/l).

Task E

Remember task A? 10,200 people have a positive faecal occult blood test result. All these people will now have a colonoscopy. The number of people who actually have colorectal cancer (500 people) is consistent. With colonoscopy all people who do not have colorectal cancer have a negative test result. 470 of those who have colorectal cancer have a positive test result.
