# Supplementary material for: Explaining computation of predictive values: 2 × 2 table versus frequency tree. A randomized controlled trial [ISRCTN74278823]
Source: BMC Med Educ. 2004 Aug 10;4:13. doi: 10.1186/1472-6920-4-13 (PMC514564; doi:10.1186/1472-6920-4-13)
Supplement: Additional File 5 — Original questionnaire used in the frequency tree group in the 2. session in German language. [file 1472-6920-4-13-S5.pdf]

Bearbeiten Sie bitte das Fallbeispiel A

# Fallbeispiel A

Für symptomfreie Frauen im Alter zwischen 70 und 79 Jahren, die im Rahmen einer Reihenuntersuchung eine Röntgenuntersuchung der Brust (Mammographie) durchführen lassen, liegen folgende Informationen vor.

**Von je 1000 dieser Frauen haben 25 Brustkrebs. Von diesen 25 Frauen, die Brustkrebs haben, erhalten 20 einen positiven Mammographie-Befund. Von den restlichen 975 Frauen, die keinen Brustkrebs haben, erhalten dennoch 97 einen positiven Mammographie-Befund.**

Abb. 1

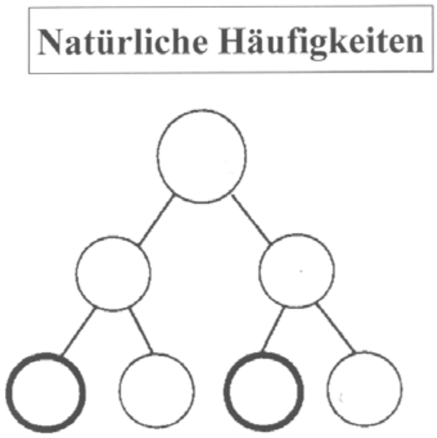


Übertragen Sie bitte nacheinander die Werte/Häufigkeiten aus dem Fallbeispiel in die dafür vorgesehenen Felder des Schaubildes (Abb.1). Ergänzen Sie der Übersicht wegen die fehlenden Werte durch Addition/Subtraktion.

**Berechnen Sie die Wahrscheinlichkeit, bei einem positiven Mammographie-Befund tatsächlich Brustkrebs zu haben.**

**Die Wahrscheinlichkeit beträgt..........%.**

Dokumentieren Sie bitte Ihre Rechenschritte.

# Fallbeispiel B

**Es wird ein HIV-Test bei 20.000 Personen durchgeführt. 300 von ihnen sind infiziert und haben dementsprechend einen positiven Befund. Bei den 19.700 Personen, die nicht infiziert waren, gab es allerdings ebenfalls 39 positive Testergebnisse.**

**Abb. 1**

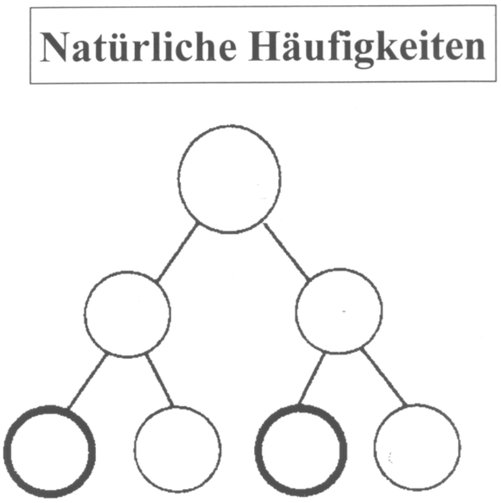


Übertragen Sie bitte nacheinander die Werte/Häufigkeiten aus dem Fallbeispiel in die dafür vorgesehenen Felder des Schaubildes (Abb.1). Ergänzen Sie der Übersicht wegen die fehlenden Werte durch Addition/Subtraktion.

**Berechnen Sie die Wahrscheinlichkeit, bei einem positiven Test tatsächlich infiziert zu sein.**

**Die Wahrscheinlichkeit beträgt.............%.**

Dokumentieren Sie bitte Ihre Rechenschritte.

**Bearbeiten Sie bitte das Fallbeispiel C**

# Fallbeispiel C

In einer luxemburgischen Studie wurde der Nutzen des Triple Tests zur Frühdiagnostik des Down-Syndroms (Trisomie 21) untersucht. Der Test wird als Bluttest im 2. Schwangerschaftsdrittel bei der Mutter durchgeführt.

**6760 Schwangere hatten an dieser Studie teilgenommen. Es gab 12 Neugeborene mit Down-Syndrom (Trisomie 21). In 9 dieser Fälle wurde dies mit dem Triple Test während der Schwangerschaft korrekterweise festgestellt. Von den übrigen 6748 Neugeborenen ohne Down-Syndrom hatten 6353 korrekterweise ein negatives Testergebnis.**

**Abb.1**

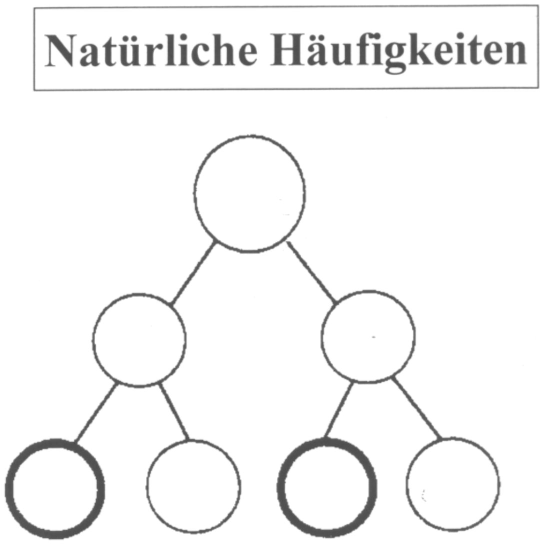


Übertragen Sie bitte nacheinander die Werte/Häufigkeiten aus dem Fallbeispiel in die dafür vorgesehenen Felder des Schaubildes (Abb.1). Ergänzen Sie der Übersicht wegen die fehlenden Werte durch Addition/Subtraktion.

**Berechnen Sie die Wahrscheinlichkeit, dass eine Schwangere mit positivem Triple Test ein Kind mit Down-Syndrom zur Welt bringt.**
**Die Wahrscheinlichkeit beträgt..........%.**

Dokumentieren Sie bitte Ihre Rechenschritte

# Fallbeispiel D

Bei 10% der Patienten mit Gallensteinen befinden sich die Gallensteine im Ductus Choledochus. Die Erkrankung wird Choledocholithiasis genannt. Um diese Erkrankung zu diagnostizieren steht als Untersuchungsverfahren, die ERCP (EndoscopicRetrogradeCholangioPancreatographie) zur Verfügung.

**200 Patienten mit Gallensteinleiden werden mit der ERCP untersucht. Von diesen 200 haben 10 % = 20 eine Choledocholithiasis. 18 dieser Patienten erhalten eine richtige Diagnose; 178 der Patienten ohne Choledocholithiasis erhalten ebenfalls eine richtige Diagnose.**

**Abb. 1**

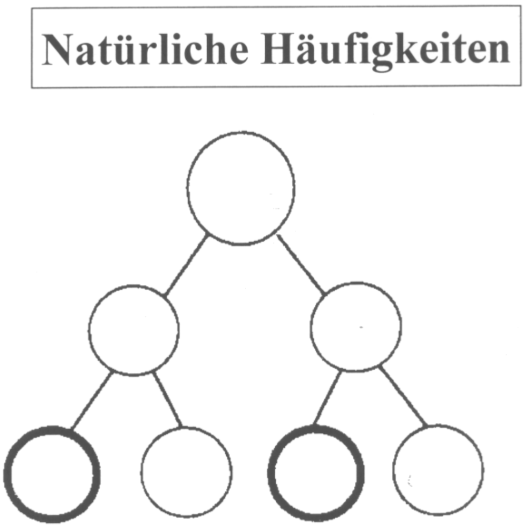


Übertragen Sie bitte nacheinander die Werte/Häufigkeiten aus dem Fallbeispiel in die dafür vorgesehenen Felder des Schaubildes (Abb.1). Ergänzen Sie die fehlenden Werte durch Addition/Subtraktion.

**Wie groß ist die Wahrscheinlichkeit einer Choledocholithiasis bei Patienten mit Gallensteinen, wenn der Untersuchungsbefund durch die ERCP positiv ausfällt?**
**Die Wahrscheinlichkeit beträgt ..........%.**

Dokumentieren Sie bitte Ihre Rechenschritte.

Bearbeiten Sie bitte das Fallbeispiel E

# Fallbeispiel E

Italienische Forscher haben herausgefunden, dass eine bestimmte Veränderung im EKG Neugeborener einen Zusammenhang mit einem später auftretenden plötzlichen Kindstod aufweist.

**Die Wahrscheinlichkeit eines plötzlichen Kindstodes liegt bei 0.07% . In einer Studie wurde bei 33.000 Neugeborenen ein EKG geschrieben. 24 von diesen 33.000 Kindern sind später an dem plötzlichen Kindstod verstorben. Die Hälfte der 24 verstorbenen Kinder hatte zuvor einen auffälligen EKG-Befund und 32.164 der gesunden Kinder hatte einen unauffälligen EKG-Befund.**

**Abb.1**

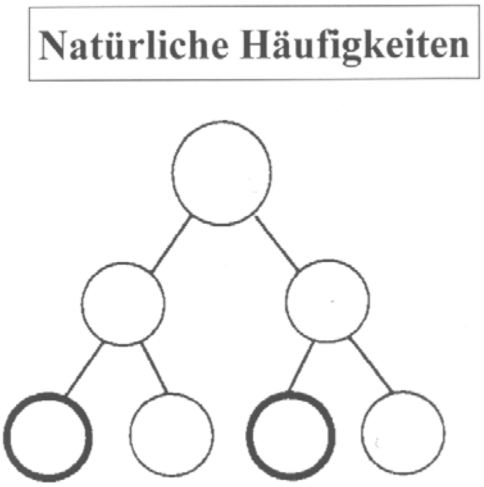


Übertragen Sie bitte nacheinander die Werte/Häufigkeiten aus dem Fallbeispiel in die dafür vorgesehenen Felder des Schaubildes (Abb.1). Ergänzen Sie die fehlenden Werte durch Addition/Subtraktion.

**Wie hoch ist die Wahrscheinlichkeit bei Neugeborenen mit positivem EKG-Befund einen plötzlichen Kindstod zu sterben.**

**Die Wahrscheinlichkeit beträgt ..........%.**

Dokumentieren Sie bitte Ihre Rechenschritte.
