# Supplementary material for: Explaining computation of predictive values: 2 × 2 table versus frequency tree. A randomized controlled trial [ISRCTN74278823]
Source: BMC Med Educ. 2004 Aug 10;4:13. doi: 10.1186/1472-6920-4-13 (PMC514564; doi:10.1186/1472-6920-4-13)
Supplement: Additional File 6 — Tasks used in the questionnaires of the follow-up examination in English language. [file 1472-6920-4-13-S6.pdf]

Tasks of the follow-up examination

The tasks of the follow-up examination are presented with the problems only.

Task A

For women aged between 70 and 79 years who participate in mammography screening the following information is available.

25 out of every 1,000 women have breast cancer. 20 out of every 25 women with breast cancer get a positive mammography. Out of the remaining 975 women without breast cancer 97 still get a positive mammography.

Task B

20,000 people take a HIV test. 300 are infected and correctly have a positive test result. 39 out of 19,700 who are not infected still had a positive test result.

Task C

6,760 pregnant women participated in a study concerning the triple test. 12 newborns have Down-syndrome (trisomy 21). 9 out of the newborns with Down-syndrome were correctly diagnosed with the test. Out of 6,748 newborns without Down-syndrome 6,353 correctly tested negative during pregnancy.

Task D

200 patients with gallstones have an ERCP (endoscopic retrograde cholangiopancreatography – a test to examine the gall duct.) 10% or 20 out of these 200 patients have gallstones in their gall ducts. 18 of these patients with gall stones in their gall ducts get a correct diagnosis. 178 patients without gall stones in their gall ducts also get a correct result.

Task E

The probability to suffer a sudden infant death syndrome (SIDS) is 0.07%. In a study 33,000 newborns have an ECG. 24 out of these 33,000 newborns die from SIDS. Half of the 24 children who died had an abnormal ECG finding and 32,164 of the surviving newborns had a normal ECG finding.
